# Unraveling access barriers and challenges of sexual and reproductive health services for individuals with psychosocial disabilities in Nigeria: insights from family, caregivers, and community stakeholders

**DOI:** 10.3389/fpsyg.2025.1562117

**Published:** 2025-08-25

**Authors:** Ibrahim Muhammad Usman, Robert A. C. Ruiter, Schaafsma Dilana

**Affiliations:** ^1^Department of Work and Social Psychology, Maastricht University, Maastricht, Netherlands; ^2^Department of Work and Social Psychology, Fontys University of Applied Sciences, Eindhoven, Netherlands

**Keywords:** access, sexual and reproductive health, psychosocial disability, community stakeholders, barriers, challenges, caregivers

## Abstract

**Background:**

Psychosocial disability (PSD) refers to the limitations experienced by persons with mental illness (PWMI) in interacting with their social environment. Persons with psychosocial disabilities (PPSD) face significant barriers to accessing sexual and reproductive health (SRH) services due to structural and institutional barriers. Despite commitments under the Convention on the Rights of Persons with Disabilities (CRPD), there are persistent rights violations and denial of PPSD to exercise their rights and access services related to SRH care. This study aimed to explore the multifaceted barriers and enablers influencing access to SRH for PPSD in Nigeria, drawing from the perspectives of caregivers, family members, and stakeholders in the communities where they live.

**Methods:**

A qualitative study method was employed, using focus group discussions (FGDs). Sixty (*N* = 60) participants were purposively and conveniently selected to include caregivers, and family members of PPSD, and community actors’ who are familiar with mental health issues and its related PSD. Responses were coded and analyzed thematically, guided by the socio-ecological framework.

**Results:**

The study reported key barriers to SRH access for PPSD, including limited mental health awareness, stigma, financial constraints, and poor healthcare infrastructure. Enablers included community awareness, financial support from informal networks, mobile outreach and staff training. Notably, the study revealed a unique culturally specific belief, such as mental illness being transmitted through breastfeeding or bringing fortune through sexual contact with affected women.

**Conclusion:**

The study highlights critical and intersecting barriers to SRH access for PPSD in Nigeria, underscoring the need for inclusive, rights-based and support system friendly interventions addressing both community-level barriers and systemic health service gaps. The study found that caregivers’ engagement and leverage of local structures within the community are essential for improving the interaction of PPSD with their physical environment and enhancing access to SRH services. Further study and SRH policy efforts must prioritize socio-cultural approaches that uphold the dignity and rights of PPSD in the community.

## 1 Introduction

Psychosocial disability (PSD) is not a disease or diagnosis but describes the limitations experienced by persons with mental illness (PWMI) when interacting with their social environment (World Health Organization, 2021). These limitations manifest in restricted functioning, social inequality, and barriers to participation in daily life, work, school, and community settings (Lovett et al., 2019). The degree of PSD varies depending on the nature of the mental condition and the surrounding social context (Starrs, 2015). The Convention on the Rights of Persons with Disabilities (CRPD) affirms the rights of persons with disabilities to equality, inclusion, dignity, and autonomy over their sexual and reproductive health (SRH) (Chandra and Chand, 2018). However, in practice, persons with psychosocial disabilities (PPSD) in Nigeria continue to face systemic and cultural barriers that hinder their ability to exercise these rights and access essential SRH services (Starrs, 2015; Issa et al., 2008).

Records from the WHO show that denial of SRH rights and services for PPSD remains a widespread public health and human rights issue, particularly in low- and middle-income countries (LMICs) in Africa and Asia that bear 88% of the global burden of mental illness (Lawal et al., 2016). In Nigeria, estimates from the Federal Ministry of Health indicate that 1.3% of the population is affected by mental illness; with PPSD experiencing more years lived with disability than those with idiopathic developmental intellectual disabilities (Yusuf and Nuhu, 2009; Coker et al., 2018). Both academic and gray literatures have reported persistent stigma, discrimination, abuse, and denial of services experienced by PPSD in Nigeria (Ukpong and Abasiubong, 2010). Anecdotal evidence from the 2020 mental health survey involving 5,484 respondents further reported these barriers and reaffirms some of the reported limitation. For instance, 69% of these respondents most of whom are caregivers of PPSD expressed unwillingness to have any form of legitimate mutual sexual relationship with PPSD, citing reasons such as illiteracy, stigma, and negative public attitudes toward PPSD as major limitations (Africa Polling Institute, 2020). Few respondents expressed conditional willingness based on economic benefit, while only 1% expressed openness to marriage (Africa Polling Institute, 2020). These attitudes reflect deeply entrenched cultural beliefs and practice that has continue to create avoidable barriers to SRH access of PPSD (Africa Polling Institute, 2020; Ayonrinde et al., 2004).

Cultural beliefs about mental illness have significantly shaped community perceptions and influenced health-seeking behaviors. In many communities, PPSD are perceived as asexual or incapable of having consensual sexual relationships, effectively excluding them from SRH considerations (Ayonrinde et al., 2004). This exclusion forms the foundation for the denial of sexual and reproductive rights, restricted access to SRH services, reduced safety and protection against sexual violence, and increased vulnerability to sexual abuse and exploitation (Ayonrinde et al., 2004; Abiodun et al., 2016).

For example, while informed consent is generally required before SRH services such as family planning can be provided, PPSD often require third-party decisions to seek health services usually from caregivers or family members who hold the right to decide when to visit the hospital and whether to consent to SRH services recommended at the facility or not (Ayonrinde et al., 2004). The decision to seek services and to give or withhold consent is mostly influenced by caregivers’ knowledge, beliefs, and values regarding mental illness and SRH (Ayonrinde et al., 2004). These social and cultural dynamics play a critical role in shaping access and contribute significantly to the ongoing denial of SRH rights for PPSD (Gureje et al., 2005; Ebuenyi et al., 2019).

These challenges are worsened by the structure and systems of mental health care delivery in Nigeria (Coker et al., 2018). Mental health care in Nigeria is largely delivered through a dual system: the conventional formal medical care provided mainly in urban and semi-urban based health facilities and community-based primary health centers (PHC) (Ayonrinde et al., 2004). The second group comprises traditional or informal care offered mainly within communities (Ayonrinde et al., 2004). Access to trained mental health care workers is extremely limited, with a ratio of one psychiatrist per 700,000 people (Lovett et al., 2019). Healthcare workers in the PHC lack adequate knowledge and training in mental health, while the majority of mental health specialists work in urban-based tertiary centers and have little engagement with community-level services (Ayonrinde et al., 2004). Mental health services are severely underfunded in Nigeria, receiving only 3.3% of the national health budget annually (Ayonrinde et al., 2004). Mental health services are regarded as specialized services and are operated through a vertical approach, with very limited integration into other services except in the urban-based tertiary health facilities (Coker et al., 2018).

This study aims to investigate the factors influencing access to SRH services for PPSD in Nigeria, focusing on the perspectives of family members, caregivers, and community stakeholders. Given their gatekeeping roles, these groups play a pivotal role in determining whether and how PPSD access SRH services. This study is part of a larger project committed to enhancing the rights and access to SRH services for PPSD in Nigeria.

## 2 Method

This study employed a qualitative design using focus group discussions (FGDs) with family members, caregivers, and community members (see Table 1). The qualitative approach facilitated the exploration of participants’ perceptions, beliefs, and experiences related to psychosocial disabilities (PSD) and access to sexual and reproductive health (SRH) services (see Appendix 2) (Palinkas et al., 2015; Glanz et al., 2008; Corbin and Strauss, 2008). Ethical approval was granted by the Ministry of Health in Sokoto State, Nigeria (SKHREC/064/2020).

**Table 1:** Participants demographics.

Variable	Category	Frequency (*n* = 60)	Percentage (%)
Gender	Male	36	60
	Female	24	40
Relationship with PPSD	Parents	12	20
	Siblings	11	18.3
	Distant relative	6	10
	Neighbor	6	10
	Civil society	12	20
	Community leader	4	6.7
	Religious leader	4	6.7
	Traditional leader	5	8.3
Educational status	Primary education	9	15
	Secondary education	18	30
	Tertiary education	33	55
Marital status	Married	48	80
	Not-married	12	20
Economic status	Gainfully employed	53	88.3
	Non-gainfully employed	07	11.7

### 2.1 Research team and reflexivity

All FGDs were conducted by the first author, a trained qualitative researcher fluent in English and Hausa. There were no prior relationships between the research team and the participants before the study. Interviews were held in participants’ preferred language (either English or Hausa) and at locations chosen for their comfort and convenience (mostly health facilities and school premises). To enhance clarity and minimize potential bias, a debriefing session was conducted to validate participants’ responses and the researchers’ interpretation of the data (see Appendix 2).

### 2.2 Setting

The research was carried out in Sokoto State, north-western Nigeria, with estimated population of 6,391,000 people and an annual growth rate of 3%. The population predominantly belongs to the Hausa and Fulani tribes and practices Islam. Family and clan structures guide social organization, with polygamy being common. Farming and livestock production dominate economic activities (80%). Poverty levels are high; with 87.7% of the population living below the poverty line and 40.1% surviving on less than $1.25 per day (National Population Commission, 2020) (see Appendix 2).

### 2.3 Participants selection

Participants (Table 1) were purposively and conveniently recruited from men and women aged 18 years and older who had direct experience living with or caring for persons with psychosocial disabilities (PPSD) within households, neighborhoods, or communities. Recruitment was facilitated through community leaders, civil society organizations (CSOs), and NGOs working in mental health. Eligibility criteria required that participants have caregiving experience or direct contact with PPSD to provide relevant insights. Of 60 participants (36 men, 24 women), ages ranged from 18 to 59 years (mean age 27.5 years). Educational backgrounds varied: 15% had primary, 30% secondary, and 55% tertiary education. Religious affiliation included 36 Muslims and 24 Christians.

### 2.4 Sample size and saturation

Ten FGDs, each with six participants, were conducted (*N* = 60). Data saturation was determined when no new themes emerged after nine FGDs, with one additional group conducted to confirm saturation (see Appendix 2) (Corbin and Strauss, 2008; McLeroy et al., 1988).

### 2.5 Data collection

Semi-structured interview guides developed from literature on SRH rights, PSD, and social cognitive theories were used to explore causes, challenges, community perceptions, and barriers to SRH services for PPSD (see Appendix 1 and 2). The socio-ecological model informed the exploration of factors at interpersonal, community, institutional, and policy levels (Palinkas et al., 2015; Glanz et al., 2008; Corbin and Strauss, 2008; McLeroy et al., 1988). FGDs lasted about 1 h and were held at convenient locations selected based on participant proximity. All sessions were audio-recorded with participant consent and transcribed verbatim. Field notes and non-verbal signs were documented during discussions.

### 2.6 Data analysis

Transcripts were imported into Atlas.ti (version 91.0) and analyzed using a combined deductive and inductive approach guided by grounded theory techniques. The steps we followed (Tables 2, 3) included data familiarization, open coding, codebook development, theme and sub-theme identification, and hierarchical organization (Corbin and Strauss, 2008; Cho and Lee, 2016). Manual transcript review supplemented software analysis to ensure comprehensive coding of factors influencing SRH access for PPSD. Selective coding synthesized codes into overarching themes. A second review was conducted on the coded data by the research team to enhance credibility and resolve discrepancies. Debriefing notes and records were utilized throughout analysis to improve trustworthiness.

**Table 2:** Summary of themes, sub-themes, codes, and supporting verbatims for barriers.

Theme	Sub-theme	Codes	Supporting verbatim
Personal-level barriers	Physical characteristics	Unusual behavior such as been nicked in open place, walking without shoes, eating from waste, isolation, restricted by others, poverty, dependency, stigma, perceived lack of value	“PPSD is a group of people with inadequate mental capacity which is affecting their rights, health status, academic performance in schools, and socioeconomic wellbeing.”
	Cause and presentation of PSD	Early-onset from mental disorders such as psychosis, late-onset from behavioral disorder such as drugs, supernatural causes from evil spirit and magic	“PSD can be caused by a lot of things, like extreme poverty, drug abuse, family history, magic attacks, and head injury.”
	Behavioral characteristics	Aggression, mood swings, irritability	“They can be calm now and become aggressive suddenly—especially when provoked or hungry.”
Interpersonal-level barriers	Relationship dynamics	Exploitation (e.g., jesters, begging), cruelty, sexual abuse risk, rejection	“Some people use them to entertain or beg. Especially the early-onset ones—people feel less empathy and treat them like tools.”
	Denial of basic rights	Incarceration, lack of mobility, exclusion from education, healthcare	“They also need to be trained through the conventional school system. Doing this can improve their standard of living”
	Lack of social support	Labeling, rejection, isolation, care burden, low expectations	“Most people believe that PPSD don‘t know anything, and cannot add any value to them.”
Organizational-level barriers	Limited services	Lack of SRH-specific services (e.g., menstrual hygiene), no trained staff, no rehabilitation centers	“Girls with mental illness don‘t get pads or even privacy. That’s when abuse can happen.”
	Health worker discrimination	Verbal abuse, class-based stigma, unequal treatment	“When a patient comes in dirty and smelling, they‘re treated like a burden.”
	Inaccessible facilities	Long distance, transport cost, user fees	“If the government can provide free care or financial support, many caregivers will not be afraid to seek help.”
	Lack of SOP/reporting	Ignorance about SOPs, no complaints mechanism	“SOP is used in government; I don‘t know about it.”
Community-level barriers	Cultural beliefs and stigma	Misconceptions, fear of hereditary transmission, spiritual attribution	“They believe if a woman with mental illness breastfeeds, the child will be mentally ill too.”
	Social exclusion	Poverty, illiteracy, shame, lack of marriage prospects	“She wears three pants so men don‘t rape her. That’s the mother’s way to protect her.”
Policy-level barriers	Lack of policy awareness	Unclear implementation of rights-based laws	“I know there’s a law that families must feed and clothe them, but I don‘t know if SRH is included.”

**Table 3:** Summary of themes, sub-themes, codes, and supporting verbatims for enablers.

Theme	Sub-theme	Codes	Supporting verbatim
Enablers to SRH access	Community support structures	Religious leaders, charity, local welfare	“We go to the imam. He prays and sometimes gives us food or money for treatment.”
	Education and awareness	Local radio, use of indigenous languages, community forums	“We heard on the radio that people with such sickness can also marry and have children.”
	Supportive caregivers	Advocacy, emotional support, accompaniment	“Her mother is always there with her, she understands and helps her get what she needs.”
	Quality of care and enabling environment	Trained workforce, respectful treatment, short wait times, outreach, hygiene, electricity	“Provide special attention for people with special needs… water, electricity, and a good toilet are a must.”
	SRH service characteristics	Free or low-cost services, mobile clinics, availability of sanitary items	“Make the service user-friendly and bring it to our area, then even the shy caregivers will come.”
	Policy suggestions	Empowerment schemes, integration of SRH, facility supervision	“Quality of care occurs when there’s healthy competition between providers. That’s when they improve their standards.”

### 2.7 Research instruments

The interview protocol contained semi-structured questions and was developed based on the empirical literature and further enriched with the help of social cognitive theories of human behavior to include the determinants of human behavior as well as the social-ecological model of health promotion (Glanz et al., 2008; Corbin and Strauss, 2008).

## 3 Results

Below we present the findings at the level of personal (cause, signs, physical characteristics), interpersonal (relationship, basic rights, network, social support), organizational (availability and quality of services, barriers, and enablers to accessing health services, standard operation procedure), and community-related (cultural practice and beliefs) factors. In addition, findings related to standard operation procedure, and channels of reporting or complaining about health workers’ attitudes are reported at the organizational level, while bylaws and policies of SRH services are reported under policy-level factors.

### 3.1 Personal-level factors

#### 3.1.1 Definition and physical characteristics of PSD

Generally, participants described PSD as a consequence of mental illness which places an affected person as less equal to a person without a mental problem. It also leads to denial of basic rights, and limitation to accessing health services. The participants described the physical characteristics of a person with mental illness as being over-dependent on others for basic needs such as access to food, health care, shelter, and protection against harm.

Others include an impaired ability to engage in productive employment leading to poverty; neglect and cruelty due to a lack of love and care from families and communities; isolation and physical restraint, which result in lost opportunities for social, educational, and economic participation, such as in schools, marketplaces, farms, or during festive periods; and abnormal reasoning, mainly due to impaired mental status.

Participant identified isolation and impaired mental status as a major cause of poverty and lack of productivity.

In addition, participants reported other physical characteristics such as irritability, aggression, hostility, and change in the usual sleep pattern. Furthermore, participants reported swinging behavior in which a person with a known mental condition can be normal at one time and aggressive at another time, usually triggered by physical provocation, hunger, drug use, and harsh living conditions such as heat and rainfall. Participants from rural communities reported that people believe having mutual sexual relations with women who have mental illness can increase wealth and fortune. Hence, many girls and women with mental illness tend to be at risk of sexual abuse.

A participant reported, *“By definition, PPSD is a group of people with inadequate mental capacity which is affecting their rights, health status, academic performance in schools, and socioeconomic wellbeing. Hence, they need support for everything.”* (A female sibling with PPSD).

#### 3.1.2 Causes of PSD

Participants classified the causes of mental illness into two broad categories based on the time of onset: early-onset and late-onset causes. Late-onset causes were identified as those typically resulting from the use of drugs, alcoholics or following major life events, such as the loss of a loved one or valuable property (e.g., a house lost to fire, theft, or business failure) which may lead to anxiety or depression. According to participants, late-onset mental illness usually begins in adulthood and is more common among men. It is often triggered by specific events, especially financial losses. Moreover, men were perceived to be more prone to substance abuse, including drugs and alcohol. In contrast, early-onset mental illness was described as typically present from birth and possibly linked to genetic factors. It may manifest with severe symptoms and is believed to affect all genders equally. Some participants, particularly those from rural areas, also attributed mental illness to supernatural causes such as black magic or evil spirits, which they saw as contributing factors to psychosocial disabilities.

A participant mentioned; *“PSD can be caused by a lot of things, like extreme poverty, drug abuse, family history, magic attacks, and head injury with impaired brain function”* (A community youth leader).

### 3.2 Interpersonal-level factors

#### 3.2.1 Interpersonal–relation

Participants have different experiences with interpersonal relationships with the PPSD. For example, some participants reported the use of PPSD as jesters to entertain guests during events or as objects used for street begging in rural communities to seek attention and to gain financial favor from pedestrians who may be sympathizing with PPSD due to their physical appearance. Not all PPSDs are qualified to be used as jesters or street begging, preferably, PPSDs with an early onset cause of mental disorders are mostly selected and addressed in a way that will catch the attention of a guest or pedestrian on the streets. Some participants reported cordial and empathic relationships including acts of almsgiving to PPSD, other respondents also reported cruelty, maltreatment, severe physical restraint, and torture of a PPSD, especially the violent type.

Participants also reported varying degrees of neglect, with some PPSD being rendered homeless, lacking both caregivers and support from family members. These categories of PPSD face significant health risks, including a higher likelihood of experiencing sexual abuse which is often unprotected, involving multiple perpetrators, and may occur concurrently. They are also at risk of violence during sexual encounters, which may result in physical injuries. In addition, homeless PPSD are at high risk of substance use, they suffer hunger, and poor personal hygiene.

#### 3.2.2 Basic human rights

The rights to live and move freely in the community were reported by the participants. In addition, the participants also condemned the incarceration and forceful isolation of persons with mental illness from other members of the community due to stigma. In addition, participants indicated the need to integrate health services for PPSD as one package; this package should protect the rights of PPSD, and promote easy access to health care, education, information, and opportunities for PPSD in the community. Participants reiterated the need to have a vanguard voice that speaks for the PPSD, especially the media or human rights groups that will fight against the rights violations that are often seen as normal due to cultural beliefs.

A participant said: *“…PPSD also need to be given rights to education and healthcare, like every citizen, they can fall sick and need health care service, they also need to be trained through the conventional school system. Doing this can improve their standard of living as well as the quality of lives…”* (a father of a 22-year-old son with PSD).

#### 3.2.3 Social support and network

Participants reported that having a mental illness is synonymous with a lack of support, lack of love, and care, and denial of rights. In addition, participants reported labeling PPSD as a lack of capacity to make a useful economic contribution to the family or the community. They are also labeled with a lack of capacity to think positively and participants raised concerns about the physical appearance of PPSD, which was reported to be a major cause of stigma among the families of PPSD within the community, which has led to a lack of interest of people in the community to associate themselves with PPSD.

Some of the participants reported as follow-up *“… PPSDs are discriminated in the community because most people believe that PPSD doesn’t know anything, and cannot add any value to them…”* (A religious leader).

Another participant reported that *“…Majority of PPSD lack the support, love, and care of their caregivers, and some people show no compassion toward them and neglect them intentionally, because taking care of them can be difficult, especially those severe and prolong types of mental illness. People feel that PPSD don’t obey instructions and don’t understand simple explanations, as a result, taking care of them can be difficult, and it is one major reason why people in the community push them away, trying to shy away from their responsibility of taking care of them. And that is why today you will see many PPSDs on the street. You may need to ask where are their caregivers, family members and friend? This alone will tell the kind of relationship PPSDs are enjoying in the communities…”* (Representative of the village head).

### 3.3 Institutional factors not organizational

#### 3.3.1 Available services

Routine maternity services such as ante and post-natal services, delivery services, family planning, and services for sexually transmitted diseases including HIV/AIDS are available. However, participants identified the need for special menstrual hygiene services and toilet training. These gaps in available services directly impact access to SRH services for PPSD.

Participants gave examples of challenges related to the lack of access to sanitary products, noting that this can increase the risk of infections and expose women and girls with mental illness during personal hygiene routines which in turn, may increase their vulnerability to sexual abuse.

In addition, participants expressed concern about the rising cases of sexual and gender-based violence (SGVB) affecting PPSD in the community, and believe that it can be addressed through comprehensive sexuality education (CSE).

They emphasized that access to SRH services must include education that equips PPSD with life skills to protect themselves from exploitation. *“…CSE is important and doable in our system but it also has to be cross-checked to ensure that the information content of the curriculum is within the acceptable limit of our culture and religion. As for abortion, I don’t think it will be accepted in the community because the teachings of our religion are not allowing it…”* (A 62-year-old Traditional ruler and leader of a community development committee). The tension between the need for CSE and cultural/religious beliefs further shapes the accessibility and acceptability of SRH services in these settings.

#### 3.3.2 Barriers to accessing SRH services

The participants reported the following as major barriers affecting PPSD from accessing SRHR services in their communities. These include poverty and financial dependency, which hinder both transportation and the ability to afford health services. Illiteracy and conceptual beliefs about mental illness were cited as root causes of stigma, which acts as a psychological and social deterrent to seeking care. The non-availability of SRH services in some communities, combined with the cost of care, long distances, and lack of transportation options, significantly reduces physical access to services. The limited number of mental health and supportive facilities in the state, including special schools and rehabilitation centers, further compounds the inaccessibility.

A participant mentioned, *“…Financial dependency of PPSD is another major challenge, but if the government can provide a system that ensures financial empowerment or free health care for PPSD that will help to address the perceived fear of the PPSD’s caregiver of cost of care that the PPSD may require…”* (A mother of an 18-year-old daughter with PSD).

These barriers demonstrate how intertwined economic limitations and structural inadequacies are in restricting SRH access. Participants also shared their encounters with health workers. The majority reported experiences of verbal and physical abuse, rejection, and discrimination, which act as powerful deterrents to accessing care.

Social hierarchy was reported to influence care, where those from affluent or royal backgrounds received preferential treatment. *“…the attitude of health workers depends on the socioeconomic status of the patient, when a patient appears with a clean dress, possibly come with personal care, they are usually treated well by health workers. But when a patient presents to the hospital in a dirty and smelling cloth, looking in rather a terrible situation, health workers treat them as a burden adds to them, not as part of their work…”* (A mother of a 12-year-old daughter with PSD).

This perception of unequal treatment further discourages marginalized PPSD from seeking SRH services.

#### 3.3.3 Enablers for accessing SRH services

Despite the barriers, participants also reported a number of enabling factors that could promote access to SRH services. These include implementing SRH services directly in the community, reducing the cost of care, and offering financial support. Strengthening the role of the ward development committee to sensitize the community and reduce stigma against PPSD was highlighted as critical. These approaches are expected to directly improve the accessibility, affordability, and acceptability of services.

A participant mentioned, *“…ensure SRH services are available and employ trained health workers to provide SRHR services in the community in a way that is effective and efficient to PPSD clients…”* (A brother to a 21-year-old sibling with PSD).

Another added, *“…Ensure that you have a system that addresses stigma and discrimination in the community and the point of service, this will help to promote access to SRH for PPSD…”* (A community member and family member of a 34-year-old female with PSD).

These reflections reinforce that service quality, staff capacity, and anti-discriminatory practices are essential to increase access. Participants described quality SRH care as including the use of modern equipment, privacy, respectful treatment, and responsive services. They reported a preference for private facilities due to the cleaner environment, better reception, and prompt service.

Public health services, by contrast, were criticized for being overcrowded and lacking basic infrastructure. *“…Quality of care occurs when there is healthy competition between service providers, usually, whenever there is a monopoly in the market the service may not be of good quality but in a situation where you want your services to speak for you as a provider you must improve in your standards…”* (A female member of a civil society organization and community women leader).

Participants further suggested that bringing services closer to the people through outreach, employing trained staff, reducing care costs, and improving facility environments will facilitate better access. *“…making the services to be user friendly. For example, provide special attention for people with special needs, reduce waiting time, and make sure other basic amenities such as water, electricity, and toilet are available…”* (Mother of a 15-year-old daughter with PSD).

Appendix Figure 1 reflects participant’s views on the quality of care including availability of clean waiting areas, electricity, and toilets were specifically noted as elements that enhance willingness to seek SRH services.

### 3.4 Community-related factors

#### 3.4.1 Cultural practice and beliefs

Participants reported a range of challenges that PPSD faced in the community, challenges ranging from restraining, torture, physical, emotional, and sexual abuse, a participant mentioned that *“…PPSD relates well with the people in the community, although with some challenges. The challenges usually come from the people in the community to the PPSD and not from the PPSD towards people in the community. Some of these challenges include physical abuse, rejection, beating, starvation, abuse, and harassment. All these challenges arise from the stigma and discrimination against PPSD in society. In a very extreme and severe culture, stigma towards PPSD for example people believe that any child that was breastfed by a mother with mental illness will develop a mental illness at a point in time, this conceptual belief usually affects their sexual and reproductive life because people are skeptical of getting married to them or from their family, this practice is still common in rural communities…”* (A brother of PPSD).

In describing the SRH challenges affecting PPSD in the community, participants mentioned a range of challenges such as stigmatization and discrimination which often precipitate and predispose other challenges and usually arise from the ignorance of mental illness or its root causes.

A participant reported as follows: *“…The first and most obvious challenge is rejection. PPSD is not accepted in the community, because there is a lack of understanding of PPSD. The way it is now, only parents with PPSD tend to accept them even because they don’t have options as we hear stories where parents dump a child with PSD and run away. Some of the reasons for the rejection include; loss of value and stigma on PPSD in the community…”* (a father of a 15-year-old girl with PSD).

In addition, participants mentioned poverty, hunger, dependency, and risk of exploitation and sexual abuse among the major challenges of PPSD.

A participant reported*, “… They are at risk of sexual abuse especially women because I know of a lady with PSD in my neighborhood who whenever she is going out her mother asked to wear 3 sets of pants for protection against sexual assault and rape…”* (women leader and member of the community development committee).

Participants further attached poverty affecting people with mental illness to lack of access to educational facilities such as schools, centers for special education, rehabilitation and vocational centers as well as centers for skills acquisition programs that can support self-reliance and give employment opportunities.

A participant said *“…Among the challenges experienced by the PPSD is financial dependency, which reduces the value of the PPSD in the community. The PPSDs don’t have money to spend in a relationship, they are not having Android or smartphones to communicate using social media, and they don’t have money to recharge their phones and call friends, loved ones, or even family. In essence, PPSD cannot socialize…”* (Youth leader in the community).

Another participant mentioned, *“…Poverty both at the individual and at the family level is another major factor affecting easy access to rights and services. We have been mentioning the cost of health care, the cost of education, and the cost involved in rehabilitation. This is not easy for a family that is not financially stable…”* (A 34-year-old male family member with a brother having PSD).

### 3.5 Organizational level

#### 3.5.1 Standard operation procedure (SOP) of providing SRH services for PPSD

In general, participants show a very shallow knowledge of standard operation procedure (SOP), and the role it has in providing clear direction and ensuring work ethics among health workers.

A participant *“…SOP is used within government system; I don’t know much about it…”* (A community leader).

#### 3.5.2 Medium to report or complain about health worker’s attitudes

Majority of the participants show no interest in reporting health workers who are behaving disrespectfully toward clients with mental illness. However, some participants mentioned the following mediums of reporting health workers for wrongdoing. Mediums listed by the participants include; the use of cell phones to call the line manager, the use of a suggestion box, and informing the head of the clinic or the unit in the facility where the incident occurs.

A participant said *“…There are several options available such as informing the health facility manager, using the suggestion box, or writing to the head of department or unit of the facility where the incidents occur…”* (A community leader and member of the ward development committee).

### 3.6 Policy level

#### 3.6.1 Policy and bye-laws

Participants show a general lack of knowledge of the policies and operating modes of the institutions either public or private. Few participants, however, reported some information about SRH policies but they are not sure whether the policy is about PPSD or not.

A participant said: *“… I know there is a policy that the government will punish any family that has a member PPSD and not providing basic needs like food, shelter, and clothing. However, I don’t know if there is a policy on SRH for PPSD…”* (a community leader and member ward development committee).

## 4 Discussion

The 10 sessions of FGD conducted were able to show the perception and level of knowledge about PPSD in the study population and also revealed key challenges, major barriers, and enablers of access to SRH service for PPSD in the study area. The challenges reported include rejection by the families, communities, and health workers at the point of seeking health service, lack of financial means to self-provide basic needs or afford health care, hunger, dependency on caregivers and family members for decision, protection, and means of livelihood, and their increased vulnerability to sexual exploitation and abuse.

These problems constituted a visual circle of continuous challenges; for example, rejection alone can lead to denials of rights to accessing services such as schools, centers for special education, rehabilitation, and vocational centers as well as centers for skills acquisition programs that are critical to self-reliance and employment opportunities leading to poverty. Poverty, in turn, causes reduced ability to afford care, and financial hardship to the family of the PPSD, poverty also causes loss of self-esteem and value in the community leading to discrimination.

Barriers reported include conceptual beliefs about mental illness, the cost of health care, the distance of the facility, stigma toward PSD (self and by association), the attitude of health workers, and the cost of transportation to the health facility. The enablers for accessing SRH services identified include; supporting the activities of the ward development committee to sensitize the community to stop discrimination against PPSD in the community. Other enablers reported include: making sure that SRH services are available in the community through mobile and outreach services, reducing the cost of care, and providing financial support to the families. In addition, maintaining trained and motivated health workforce, and conducting regular facility supervision visits by the team of supervisors from the Ministry of Health.

When compared to existing literature, these findings are consistent with studies in similar under-resourced settings that report stigma, poverty, and weak health systems as critical barriers to SRH access for persons with mental illness (Yusuf and Nuhu, 2009; Coker et al., 2018; Ukpong and Abasiubong, 2010; Africa Polling Institute, 2020; Ayonrinde et al., 2004). However, the specific cultural perceptions reported in this study, such as beliefs around acquiring mental illness through breastfeeding and the potential increase of wealth and fortune through mutual sexual relations with women having mental illness, these highlights unique geographic and culturally specific barriers commonly found in LIMCs. In high-income settings, policies and structured social welfare systems often play a protective role for PPSD, whereas in LIMC settings like the study area, low literacy levels about mental health and SRH influence the interactions of caregivers, family, and community members toward PPSD, leading to systemic neglect and abuse.

These findings confirm the existence of disability due to mental illness during interactions with the immediate environment. Stigma and discrimination reported among health care workers and the lack of an institutionalized system of protective mechanisms also add important context-specific insights into how geographic, cultural, and systemic differences influence access to SRH services.

The study was able to reveal the influence of socioeconomic impact on access to SRH services by PPSD due to over-reliance on others for financial support. In addition, the study also revealed poor knowledge of standard operating procedure (SOP), poor understanding of quality of care related to SRH services, and limited awareness of the available channels for reporting the wrong attitude of health workers among major stakeholders supporting PPSD to access SRH services. Other barriers reported in this study, is distance to health facilities, especially for rural settlers, and financial burden particularly because a companion or accompanying person is required to go with PPSD, as they cannot go alone. These barriers indicate both financial and in self-independence disabilities of PPSD in accessing SRH services.

The study provided detailed and specific information about the nature of the relationship between PPSD and the community by confirming the excessive, conscious, and deliberate actions of some family members and the community to exploit persons with mental illness financially by engaging them as jesters or street beggars. The need for special training on menstrual hygiene and toilet training as well as easy access to sanitary packs for women and girls with mental illnesses confirmed that the SRH requirement for PPSD is gender-specific. Hence, the study reported unhygienic menstrual practices and their potential effect on the fertility of PPSD.

The study also reported common but archaic practices such as severe physical restraint, and torture of a person with mental illness, especially the violent type. The study highlighted the predisposing factors to the aggressive or violent mood of PPSD caused by swinging mood disorder which is usually triggered by physical provocation, hunger, and harsh living conditions such as heat and rainfall. All these triggers have a better remedy than physical restraints in chains or incarceration for PPSD.

The study underscores the importance of tailored interventions to educate families, caregivers, and communities about the rights of PPSD and to fight against discrimination. Participants highlighted gaps and challenges that require targeted policy interventions to address persistent barriers such as stigma, financial burden, and limited protection of the rights and safety of PPSD. This finding aligns with best practices reported in the literature from HICs (Coker et al., 2018; Ukpong and Abasiubong, 2010; Africa Polling Institute, 2020; Ayonrinde et al., 2004). Community interventions such as public awareness and campaign strategies, mobile, and outreach health services have been identified to improve access to SRH services for PPSD. The study indicates the crucial need to address gender-specific needs such as menstrual hygiene.

Lastly, the study findings show several limitations. For example, the study was unable to establish the extent to which these factors can guarantee easy access to quality SRH services for PPSD in the community; hence, further study in this area is recommended. The study findings were based on what the participants knew or had experienced in the past; hence, the risk of recall bias is eminent. However, we used COREQ (Appendix Table 4) to improve reliability and reduce the risk of bias. Participants were also reluctant to share their experiences or perceptions on sensitive issues such as safe abortion services and rape. However, they were reassured of their anonymity in the research. The study’s major strength is the use of FGDs, which allowed participants to build on the ideas of fellow participants.

## 5 Conclusion

Persons with psychosocial disabilities (PPSD) have equal rights to bodily autonomy, reproduction, and access to sexual and reproductive health (SRH) services. However, due to their mental condition, they often rely on support from family, caregivers, and the community. This study identifies several barriers to SRH access for PPSD, including stigma, risk of abuse, denial of rights, and sociocultural beliefs. It also highlights enablers such as poverty reduction, affordable care, and community sensitization through ward development committees. The findings offer context-specific insights from under-researched settings, emphasizing the need for locally informed policies and interventions in mental health and SRH.

## Data Availability

The original contributions presented in the study are included in the article/Supplementary material, further inquiries can be directed to the corresponding author.
