# Supplementary material for: Unraveling access barriers and challenges of sexual and reproductive health services for individuals with psychosocial disabilities in Nigeria: insights from family, caregivers, and community stakeholders
Source: Front Psychol. 2025 Aug 25;16:1562117. doi: 10.3389/fpsyg.2025.1562117 (PMC12414784; doi:10.3389/fpsyg.2025.1562117)
Supplement: Supplementary file 1 [file Supplementary_file_1.docx]

*Appendix*


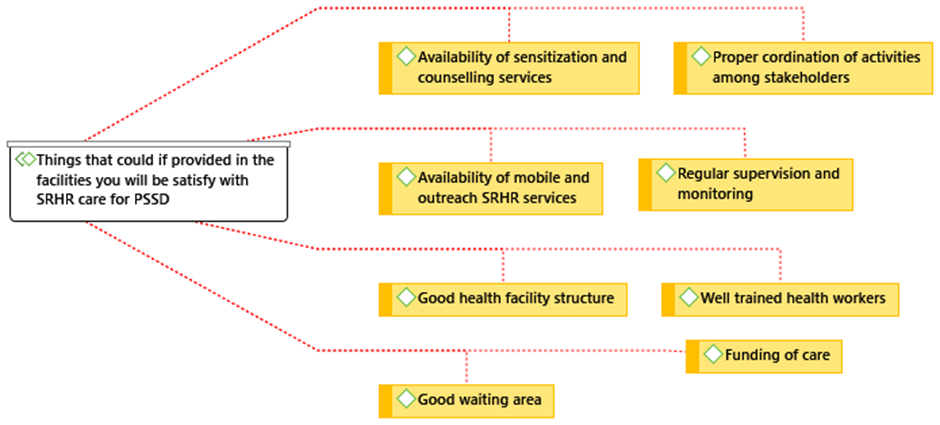


Fig.1: Promoters of good quality SRH care for PPSD

Table 4: CHOREQ Check-list

| **COREQ Domain** | **Item** | **Description** | **The Study** |
| --- | --- | --- | --- |
| Team/Reflexivity | Interviewer/facilitator | Who conducted the interviews or FGDs? | The first author conducted all FGDs. |
|  | Credentials | Researcher’s qualifications | The first author is a trained qualitative researcher fluent in English and Hausa. |
|  | Experience and training | Relevant experience/training of interviewer | Experienced in qualitative methods and mental health research. |
|  | Relationship established | Relationship with participants prior to study | No prior relationship, minimizing bias. |
|  | Participant knowledge of interviewer | Participants were aware of researcher’s background, goals | Informed consent included explanation of study goals; rapport built during FGDs. |
|  | Reflexivity | Steps taken to address researcher bias and assumptions | Reflexivity maintained via writing and debriefing sessions. |
| Study Design | Methodological orientation and theory | Theoretical framework guiding study design | Socio-ecological model and social cognitive theories. |
|  | Sampling | Sampling method | Purposive sampling through community leaders, CSOs, and NGOs. |
|  | Method of approach | How participants were approached | Through community networks and NGOs active in mental health. |
|  | Sample size | Number of participants | 60 participants (36 men, 24 women) in 10 FGDs. |
|  | Non-participation | How many refused or dropped out | Not specified; all recruited participants consented and participated. |
|  | Setting | Where data were collected | Sokoto State, Nigeria; FGDs held at convenient, private locations. |
|  | Duration | Length of interviews or FGDs | Approximately one hour per FGD. |
|  | Data saturation | Was data saturation discussed? | Data saturation reached after 9 FGDs, confirmed by 10th FGD. |
| Data Collection | Interview guide | Use of guides | Semi-structured guide developed from literature and theory (see appendice1). |
|  | Repeat interviews | Were repeat interviews carried out? | No. |
|  | Audio/visual recording | Were FGDs audio recorded? | Yes, all FGDs audio recorded with participant consent. |
|  | Field notes | Were field notes taken? | Yes, including non-verbal cues. |
|  | Transcripts returned | Were transcripts returned to participants for comment/correction? | Not specified. |
| Data Analysis and Reporting | Number of data coders | How many researchers coded the data? | Two coders involved in coding and theme validation. |
|  | Description of the coding tree | Was the coding tree or scheme described? | Coding scheme developed inductively; hierarchy of themes and sub-themes described. |
|  | Software | Software used | Atlas.ti version 91.0. |
|  | Participant checking | Were participants asked to verify findings? | Not specified. |
|  | Quotations presented | Were participant quotations used to illustrate themes? | Quotations are included (per overall manuscript, though not in method section). |
|  | Consistency of data and findings | Consistency between data presented and findings | Themes and sub-themes clearly linked to coded data and quotes; second coder confirmed coding. |
|  | Clarity of major themes | Are major themes clearly presented? | Yes, themes organized and presented clearly. |
